# Serum adropin level in wet-type age-related macular degeneration

**DOI:** 10.1186/s40942-024-00543-7

**Published:** 2024-03-12

**Authors:** Zahra Saeedi-Maleki, Alireza Javadzadeh, Fariborz Brumandpur, Amir Ghorbanihaghjo, Shokoufeh Khanzadeh, Farideh Mousavi

**Affiliations:** 1 Department of Ophthalmology, Tabriz University of Medical Sciences, Tabriz, Iran; 2 Biotechnology Research Center, Tabriz University of Medical Sciences, Tabriz, Iran; 3 Drug Applied Research Center, Tabriz University of Medical Sciences, Tabriz, Iran; 4 Tabriz University of Medical Sciences, Tabriz, Iran

**Keywords:** Adropin, Age-related macular degeneration, High-Density Lipoprotein

## Abstract

**Purpose:**

Our objective was to compare the serum Adropin levels between patients with wet-type Age-Related Macular Degeneration (AMD) and otherwise healthy individuals.

**Method:**

The study included 45 patients with wet-type AMD and 45 individuals without age-related macular degeneration. Patients with co-morbidities such as diabetes, hypertension, autoimmune diseases, and a previous history of visual impairment; were excluded. FBS, Hemoglobin A1C (HbA1C), lipid profile, and serum Adropin level were checked.

**Results:**

The mean serum Adropin level of patients with wet-type AMD was significantly lower than the control group (P-value < 0.001). Also, the mean High-sensitivity C-reactive protein ( hsCRP) level and High Density Lipoprotein (HDL) were significantly higher in wet-type AMD patients (P-value = 0.031 and < 0.001 respectively).

**Conclusions:**

In our study, wet-type AMD was associated with a lower level of serum Adropin. Because of Adropin involvement in glucose metabolism and age-related changes, it may have a role in the pathogenesis of AMD, but it requires more investigations at the molecular level to elucidate its function.

**Supplementary Information:**

The online version contains supplementary material available at 10.1186/s40942-024-00543-7.

## Introduction

Age-related macular degeneration (AMD) is one of the most common causes of visual impairment globally. It is a chronic and progressive disorder that affects the choroid, sub-retinal Pigment Epithelium (RPE), and sub-retinal layers; and it is predicted that 288 million individuals will be affected by AMD by the year 2040 [1]. AMD is distinguished by its clinical manifestations such as the presence of drusen and irregularities in the RPE layer at its early stage. Late-stage AMD is defined in two forms: the first type is neovascular, also known as wet or exudative AMD; and the second type is non-neovascular, also known as geographic atrophy [2].. Late-stage AMD causes a decrease in central visual acuity, leading to severe and irreversible vision loss and legal blindness, severely compromising the patient’s quality of life and functional independence [3]. In neovascular AMD, choroidal neovascularization occurs when new blood vessels grow from the choroid layer, penetrating the RPE and Bruch’s membrane. This can cause the leakage of fluid, lipids, and blood; eventually leading to the formation of fibrous scars [4] Around 10% of individuals diagnosed with AMD may experience the complications of wet-type AMD. Multiple significant risk factors are implicated in the pathogenesis of AMD which are including smoking, dietary factors, cardiovascular disorders, the complement regulator Factor H, lipids, angiogenetic agents, and the products of extracellular matrix pathways. Aging is the main and most important risk factor for AMD. The relevance between AMD and female sex as a risk factor has shown inconsistent findings in various research [5]. The current approach to managing neovascular AMD involves the inhibition of Vascular Endothelial Growth Factor (VEGF) which is an angiogenetic protein. VEGF is naturally produced in the retina while it can be induced by conditions such as ischemia and inflammation [6]. Moreover, recent studies have elucidated the key role of VEGF in choroidal neovascularization. VEGF is the predominant and most significant contributor to angiogenesis in patients with AMD among various angiogenetic factors [7, 8]. There are two main VEGF receptors which are tyrosine kinase receptors, designated as vascular endothelial growth factor receptor-1 (VEGFR-1) and vascular endothelial growth factor receptor-2 (VEGFR-2) [9]. By blocking VEGF action, it is possible to reduce retinal vascular permeability; thereby preventing fluid leakage, and also to hinder the formation of abnormal blood vessels (neovascularization) in the retina [10].

Adropin, a peptide consisting of 76 amino acids, was initially introduced in 2008. It is encoded by the energy homeostasis-associated gene (Enho) and is primarily found in tissues characterized by high metabolic activity, including the brain and the liver [11]. Adropin has also been detected in various peripheral organs, including the heart, lungs, the medulla of kidneys, muscles, peripheral blood mononuclear cells, and breast cancer cells [12, 13]. Adropin is involved in energy homeostasis, metabolic adaptation to macronutrients, and modulation of insulin sensitivity and diabetes [14]. Recent studies have suggested that Adropin might have additional functions beyond metabolism, such as controlling vascular endothelial function [15]. This protein is involved in the regulation of various metabolic activities, including the formation of new blood vessels (angiogenesis) and the preservation of optimal blood flow within the cardiovascular system [16] Adropin also upregulates VEGFR-2 on the surface of endothelial cells, which causes antiangiogenic activity [9]. Recent studies have provided evidence of the protective effects of Adropin on cerebral endothelial cells under conditions of hypoxia and low glucose. Adropin has been shown to reduce vascular permeability and leakage in these circumstances. Adropin has a key role in regulating lipid accumulation, often in highly specialized cells such as the retinal tissue, and acts as a modulator of the fatty acid oxidation (FAO) pathway. The energy metabolism in retinal cells relies heavily on FAO for energy production. However, aerobic glycolysis is primarily utilized for synthesizing phospholipids in the outer segments of the retina [17]. Here in this article, we conducted a case-control study evaluating serum Adropin levels in patients diagnosed with wet-type AMD in comparison with the normal population.

### Method

This case-control study was accomplished in the Nikookari Hospital and Sheykholrais Laboratory, Tabriz, Iran from January 2022 to July 2022. Participants referred to Nikookari Hospital (a referral specialized eye center in northwest Iran) and diagnosed with wet-type AMD were included. Ethical approval was obtained from the Institute of Ethics Committee of Tabriz University of Medical Sciences with a code number of IR.TBZMED.REC.1400.981, and an informed written consent form was obtained from all the participants in the study. All data of patients will remain confidential and the study protocol complies with the declaration of Helsinki.

### Study protocol

This study consisted of two groups, 45 in each. Group A included wet-type AMD patients (Cases) and Group B included 45 normal healthy individuals (Controls) which were selected to be similar in terms of age, sex, and body mass index (BMI). One of the samples in the case group was lost due to clotting. A complete ophthalmic examination including best corrected visual acuity (BCVA) (based on LogMAR) and slit lamp examination was done by an ophthalmologist.

After identifying and selecting the individuals to participate in this study, all of them were referred to the Sheykholrais laboratory (Tabriz University of Medical Science Laboratory) for venous blood sampling. Blood samples were taken after overnight fasting. After collecting 5 ml of venous blood samples which were immediately centrifuged, we stored serum samples at -70 ° C for biochemical analysis. Laboratory specialists were blinded in this study.

Measurement of Adropin was done by ELISA method (ZellBio GmbH, Germany) using primary and secondary antibodies, adding the substrate, measuring the corresponding wavelength, and utilizing a standard curve according to the instructions of the corresponding kit. Measurement of High sensitivity C-reactive protein (hs-CRP) as an inflammation marker was performed by an immunoturbidimetry method using an auto-analyzer, by utilizing polyclonal antibody and measuring changes in light absorption. Serum levels of fasting blood sugar (FBS), hemoglobin A1c (Hb A1c), total cholesterol (Chol), triglyceride (TG), high-density lipoprotein cholesterol (HDL), low-density lipoprotein cholesterol (LDL) were determined by using commercial kits (Pars Azmoon, Tehran, Iran) with an automated chemical analyzer (Roche Hitachi 917 Rack Chemistry Analyzer, Basel, Switzerland).

### Inclusion criteria

Patients with the diagnostic criteria for wet-type AMD in the case group such as bilateral changes in the macula including the presence of drusen, macular atrophy, and pigmentary changes, along with the choroidal neovascularization at least in one eye, who attended Nikookari hospital without any previous history of visual impairment, were included.

### Exclusion criteria

Individuals with underlying disorders such as diabetes, hypertension, BMI > 30, patients with a history of chronic or acute infections, autoimmune diseases, chronic liver and kidney diseases, seizures, and bleeding disorders; were excluded from our study. Also, patients with a history of substance abuse, and a history of pre-existing visual impairment were excluded.

### Statistical analysis

The participants’ demographic data (Gender, age, BMI) and the outcome variable data (the level of serum Adropin, hs-CRP, FBS, HbA1c, TG, Chol, HDL, and LDL) were expressed as mean ± standard deviation or median with interquartile range, as appropriate for the type of the distribution. Comparisons of the data were performed using an independent t-test or Mann-Whitney U test as appropriate. Also, the chi-square test was used to compare the two-level or multi-level parameters between the case and control groups. P value < 0.05 is considered statistically significant. SPSS version 26 was used for all statistical analysis.

## Results

Ninety participants were initially included in this study. One participant was excluded from the study due to the loss of the serum sample, and the analysis was performed on 44 and 45 individuals in the case and the control groups respectively. The comparison of demographic parameters between the case and control groups is shown in Table 1. There was no significant difference between gender (*P* = 0.167), and age (*P* = 0.102) of the case and the control groups.

**Table 1:** Demographic data between two groups

Parameters		Cases (*n* = 44) n (%)		Controls (*n* = 45) n (%)	P
Gender	Male	26(59.1)		20(44.4)	.167^a^
	Female	18(40.9)		25(55.6)	
Age (years); mean (SD)		72.43(6.82)		70.29(5.31)	.102^b^
BMI; mean (SD)		27.41(4.30)		27.55(4.62)	.83b
RE BCVA (LogMAR); mean (SD)		0.97(0.64)		0.13(0.31)	<.001^b^
LE BCVA (LogMAR); mean (SD)		1.18(0.58)		0.20(0.40)	<.001^b^

a: Chi-squared test, b: Independent t-test;

RE BCVA: Right Eye Best Corrected Visual Acuity, LE BCVA: Left Eye Best Corrected Visual Acuity, LogMAR: Logarithmic Minimum angle of resolution, BMI: Body Mass Index

The comparison of the main parameters between the two groups is shown in Table 2. There wasn’t any statistically significant difference observed among parameters of FBS(*P* = 0.097), HbA1c (*P* = 0.238), TG (*P* = 0.691), Chol (*P* = 0.563), and LDL (*P* = 0.992) between the two groups. The results showed that the mean serum level of Adropin among wet-type AMD patients was significantly lower compared to the control group (*P* < 0.001). Also, the mean hs-CRP (*P* = 0.031) and the serum HDL level (*P* < 0.001) were at a higher level in wet-type AMD patients in comparison with the control group.

**Table 2:** Assayed parameters of study participants among the two groups

Parameters	Cases (*n* = 44) n (%)	Controls (*n* = 45) n (%)	P
Adropin (Pg/mL)	268.75(257.97-284.52)	331.00(317.70-341.65)	<.001^b^
hsCRP (mg/L)	4.20(3.62–5.65)	3.70(3.10–4.55)	.031^b^
FBS (mg/dL)	102.50(92.00-115.00)	93.00(87.00-99.50)	.097^b^
HbA1C(%)	5.74(0.70)	5.58(0.58)	.238^a^
TG (mg/dL)	128.00(98.50-158.75)	127.00(98.50–186.00)	.691^b^
Chol (mg/dL)	192.64(48.90)	186.80(45.87)	.563^a^
HDL (mg/dL)	54.75(9.99)	46.87(8.62)	<.001^a^
LDL (mg/dL)	110.55(38.47)	110.63(34.44)	.992^a^

a: Mann–Whitney U test (Data were expressed in terms of median with interquartile range); b: Independent t-test, hsCRP: high sensitive C-reactive protein, FBS: fasting blood sugar, HbA1C: Hemoglobin A1C, TG: Triglyceride, Chol: Cholesetrol, HDL: high density lipoprotein, LDL: low density lipoprotein

**Table 3:** Correlation among study parameters within cases

total cholesterol Vs HDL, LDL, TG						
Cases (*n* = 44)	HDL (mg/dL)		LDL (mg/dL)		TG (mg/dL)	
	r	p	r	p	r	p
Total cholesterol (mg/dL)	0.617	< 0.001	0.960	< 0.001	0.436	0.003

Data were expressed by using Spearman’s rank correlation test: *P-value < 0.05 - considered as statistically significant: HDL: High-density lipoprotein cholesterol, LDL: Low-density lipoprotein cholesterol, TG: Triglyceride

## Discussion

Adropin plays a role in both energy metabolism and age-related systemic changes. Therefore, we aimed to evaluate its association with AMD. Erman et al., showed that age is the most effective factor affecting serum Adropin levels. Also, lower concentrations were observed in obese patients. Serum Adropin levels were negatively correlated with BMI, FBS, and fasting insulin concentration [18]. Therefore, in this study, by matching age and gender in two groups and eliminating other confounders such as Diabetes and BMI > 30, we tried to reduce the effect of these factors on the serum level of Adropin. One of the important goals of our research was to investigate any possible association between Adropin levels and wet-type AMD, which had not been discussed in recent studies as far as we know.

We evaluated 89 individuals and categorized them sin two groups containing 44 patients diagnosed with wet-type AMD in the first group and 45 normal healthy people in the second group. The demographic data of the two groups was assessed, and relevant biochemical test data was collected. These data included serum levels of Adropin, hs-CRP, FBS, HbA1c, and lipid profile.

In this study, the comparison of Adropin levels in two groups showed lower Adropin levels in wet-type AMD patients, and since the decrease in serum Adropin levels is associated with vascular endothelial dysfunction, this can be the cause of this finding. This is against the findings of the study by Ornek et al., who found that the wet-type AMD group had insignificantly (*P* = 0.07) higher serum levels of Adropin as a response to pathological angiogenesis or as an early sign of endothelial dysfunction and atherosclerosis [10].

According to our study, the case group’s serum HDL levels were higher, which was in line with Wang et al.‘s systematic review study, which found a statistically significant increase in the risk of AMD related to higher serum HDL levels. Also, some research discovered that while greater levels of Chol, LDL, or TG are linked with a lower risk of AMD, particularly in its early stages; higher levels of HDL may increase a person’s chance of developing AMD. Furthermore, several genes that enhance HDL-C levels within the HDL-C pathway, including the lipoprotein lipase gene, the cholesterol ester transferase (CETP) gene, and the ABC-binding cassette A1 gene; have been identified as being correlated with an elevated susceptibility to AMD [19]. A positive association between total cholesterol levels and HDL, LDL, and TG was discovered in individuals with wet-type AMD. This outcome matched those made by Neethu et al. in their investigation. The action of Adropin on retinal lipid metabolism may thus affect the pathogenesis of lipid-mediated AMD, which has long been hypothesized to include abnormal lipid metabolism [20]. On the other hand, Burgess et al. declared that variants in the CETP gene region associated with increased circulating HDL-cholesterol also associated with increased AMD risk, although variants in the LIPC gene region that increase circulating HDL-cholesterol have the opposite direction of association with AMD risk [21]. So it seems that any increase in the serum HDL levels is not the cause of AMD and other functioning areas of these genes may be involved in the development of AMD.

The serum level of hs-CRP in this study was significantly higher in the case group. This finding aligns with the results reported in a systematic review conducted by Mitta et al. they have found that a single measurement of hs-CRP more than 3 mg/L predicts an increased risk of developing.

AMD over many years. Also individuals with baseline hsCRP levels more than 3 mg/L had a 50% increased risk of incident AMD and a nearly 2-fold increased risk of wet-type AMD [22].. Additionally, given that Adropin levels fall in inflammatory disorders, the low levels of Adropin found in these individuals may imply that AMD is an inflammatory condition, and increased serum concentration of hs-CRP may be evidence for this argument. Additionally, it was suggested in research by Ornek et al. that decreasing serum VEGFR levels could be connected to the development of AMD. Clinical trials have demonstrated a connection between endothelial dysfunction and the development of drusen in AMD patients, and also the progression of AMD. Adropin appears to have a role in the pathogenesis of age-related disorders such as wet-type AMD due to its functions in hemostasis, angiogenesis, regulation of inflammatory responses, regulation of fat storage, and an antioxidant effect [16].

According to the few studies that have been conducted in this case, more studies are required in this field. On the other hand, investigating the relationship between the serum level of Adropin and the risk of wet-type AMD may provide us with useful information about the more precise pathogenesis of this disease, which can be a useful step toward the prevention or treatment of this debilitating disease.

We had some limitations in our study. First of all, the study groups were small which can narrow the results of analysis. Second, we investigated the individuals of just one specialized eye center, without regarding the genetic and environmental parameters affecting the patient and control groups.

## Conclusions

These results suggest that AMD may be associated with lower Adropin levels, and Adropin may play a role in the pathogenesis of wet-type AMD, but to clarify this, we need to conduct investigations at the molecular level. Increased serum HDL levels were observed among patients with wet-type AMD but because of the variety of genes involved in HDL production, this can be only an association, not the causal effect of HDL in the pathogenesis of wet-type AMD.

### Electronic supplementary material

Below is the link to the electronic supplementary material.

Supplementary Material 1

Supplementary Material 2

## Data Availability

The datasets used and/or analysed during the current study are available from the corresponding author on reasonable request.

## Abbreviations

AMD: Age Related Macular Degeneration
BCVA: Best Corrected Visual Acuity
BMI: Body Mass Index
CETP: Cholesterol Ester Transferase
Chol: Cholesetrol
FAO: Fatty Acid Oxidation
FBS: Fasting Blood Sugar
HbA1C: Hemoglobin A1C
HDL: High Density Lipoprotein
hsCRP: High-sensitivity C-reactive protein
LDL: Low Density Lipoprotein
LE: Left Eye
LogMAR: Logarithmic Minimum angle of resolution
RE: Right Eye
RPE: Retinal Pigmented Epithelium
TG: Triglyceride
VEGF: Vascular Endothelial Growth Factor
VEGFR: Vascular endothelial growth factor receptor
