# Supplementary material for: Serum adropin level in wet-type age-related macular degeneration
Source: Int J Retina Vitreous. 2024 Mar 12;10:27. doi: 10.1186/s40942-024-00543-7 (PMC10935851; doi:10.1186/s40942-024-00543-7)
Supplement: Supplementary file 1 — Supplementary Material 1 [file 40942_2024_543_MOESM1_ESM.docx]

Dear Chief Editor of IJRV magazine

I hope this email finds you well. I have to thank you for making an exception in reviewing our article (Serum Adropin level in wet-type age-related macular degeneration) in your magazine. I want to inform you about the exact part of Tabriz University of Medical Sciences has funded this article. I am going to change the fund part in the revised article as below:

Fund: Drug Applied Research Center, Tabriz University of Medical Sciences, Tabriz, Iran.

Also, dr. Shokoufeh Khanzadeh has helped us in the writing of this article. So if you agree, I will add her name as the 6^th^ writer with this affiliation:

Tabriz University of Medical Sciences, Tabriz, Iran.

The revised article will be ready before the 14^th^ of December as you asked.

Kind regards

Dr. Mousavi
