# Supplementary material for: Serum adropin level in wet-type age-related macular degeneration
Source: Int J Retina Vitreous. 2024 Mar 12;10:27. doi: 10.1186/s40942-024-00543-7 (PMC10935851; doi:10.1186/s40942-024-00543-7)
Supplement: Supplementary file 2 — Supplementary Material 2 [file 40942_2024_543_MOESM2_ESM.docx]

Dear editor

I hope this email finds you well. I have noticed that the number 9 reference is not validated by the journal. I provided the link through google scholar to double check it

The link for mentioned article is:

<https://scholar.google.com/scholar?hl=en&as_sdt=0%2C5&q=9.+%C3%96rnek+N%2C+%C3%96rnek+K%2C+Aydin+S%2C+Yilmaz+M%2C+%C3%96lmez+Y.+Serum+vascular+endothelial+growth+factor+receptor-2+and+adropin+levels+in+age-related+macular+degeneration.+Int+J+Ophthalmol.+2016%3B+9%284%29%3A+p.+556-60.&btnG=>

and the citation by the Vancouver method is as below:

Örnek N, Örnek K, Aydin S, Yilmaz M, Ölmez Y. Serum vascular endothelial growth factor receptor-2 and adropin levels in age-related macular degeneration. International Journal of Ophthalmology. 2016;9(4):556.

I am looking forward to hearing from you.

Kind regards

Dr. Mousavi
